# Real-time assessment of circulating tumor cells refines the indication for HER2-targeted therapy in metastatic gastric cancer

**DOI:** 10.1038/s41598-025-06913-x

**Published:** 2025-07-04

**Authors:** Yasuaki Kimura, Koichi Suzuki, Sawako Tamaki, Iku Abe, Yuhei Endo, Kosuke Ichida, Yuta Muto, Fumiaki Watanabe, Masaaki Saito, Kazuo Takeda, Toshiki Rikiyama

**Affiliations:** 1 Department of Surgery, Saitama Medical Center, Jichi Medical University, 1-847, Amanuma-cho, Omiya-ku, Saitama, 330-8503 Japan; 2 On-chip Biotechnologies Co., Ltd, Tokyo, Japan

**Keywords:** Advanced gastric cancer, Circulating tumor cells, HER2, Liquid biopsy, Gastric cancer, Prognostic markers, Tumour biomarkers, Tumour heterogeneity

## Abstract

HER2-targeted therapies have improved outcomes in metastatic gastric cancer (mGC), yet assessment of HER2 status in tumor tissues remains limited by heterogeneity and temporal changes. This study aimed to evaluate real-time HER2 expression on circulating tumor cells (CTCs) using the On-chip Sort system. CTCs were enriched from blood samples of 27 mGC patients, identified by cytokeratin staining, and assessed for HER2 expression via fluorescent labeling. The epithelial-mesenchymal transition (EMT) index was calculated based on co-expression of vimentin and cytokeratin. CTCs also underwent whole-genome amplification and targeted sequencing using a cancer gene panel. Patients were stratified into three groups: Group A (*n* = 13), HER2-positive in tissue; Group B (*n* = 8), tissue HER2-negative but CTC HER2-positive; and Group C (*n* = 6), HER2-negative in both tissue and CTCs. All patients received cytotoxic chemotherapy; only Group A received additional HER2-targeted therapy. Group B showed the poorest progression-free survival (PFS: 7.0 months), compared to Group A (15.7 months) and Group C (not reached). CTC HER2 expression correlated with EMT index; Groups A and B also exhibited higher EMT indices and shared EMT-related mutations. These findings suggest that CTC-based HER2 monitoring reflects tumor aggressiveness and may help identify patients who could benefit from HER2-targeted therapy despite negative tissue HER2 status.

**Supplementary Information:**

The online version contains supplementary material available at 10.1038/s41598-025-06913-x.

## Introduction

Gastric cancer (GC) is one of the most common cancers and the third leading cause of cancer-related deaths worldwide; more than one million patients worldwide are diagnosed with GC each year^1,2^. Currently, the primary therapy for patients with GC is surgery and systemic chemotherapy, while radiotherapy, immunotherapy, and targeted therapy are gradually being included^1,3–5^. Several treatment and diagnostic improvements have been achieved in the last few decades, although the prognosis remains poor for advanced GC^6^. Four molecular subtypes of GC have been identified^7^, with the subgroup overexpressing human epidermal growth factor receptor 2 (HER2) seeing major improvements in therapies^1,7,8^.

Overexpression or amplification of HER2 has been observed in 15–20% of patients with advanced GC^9–14^. Patients with HER2-positive tumor harbor more aggressive features, resulting in a poorer prognosis^8,14–16^; hence, they require treatment with appropriate interventions. In patients with HER2-positive advanced GC, the Trastuzumab for Gastric Cancer (ToGA) trial demonstrated that the addition of trastuzumab, an anti-HER2 monoclonal antibody, to chemotherapy was significantly beneficial^17^. Despite the success of HER2-targeted therapies in HER2-positive patients with GC, the conventional assessment of HER2 status in tumor tissue specimens remains debatable. GC harbors temporal, spatial, and intertumoral heterogeneities^18,19^, which may provide insufficient clues for the assessment of HER2 status and selection of potential candidates for HER2-targeted therapies^20^. The genomic profiles of primary tumors and metastases are not always concordant, and metastatic tumor tissues are difficult to obtain^21,22^. To provide appropriate treatment options for these potential HER2-positive patients with GC, it is critical to develop better strategies for real-time assessment of tumor dynamics.

A blood-based technology platform that tracks circulating tumor DNA (ctDNA) and circulating tumor cells (CTCs), known as liquid biopsy, offers an option for noninvasive monitoring of therapeutic responses in various cancers^23–28^. Detection of HER2 amplification in ctDNA assists guided selection of patients suitable for HER2-targeted therapies. HER2 copy number monitoring in ctDNA was reportedly beneficial for identification of HER2 positive conversion, in recurrent tumors of patients who did not have HER2 amplification in primary tumors^22^. However, DNA to protein syntheses are regulated at transcription and translation checkpoints^29^, which may result in potential negative expression of HER2 protein in cancer cells with HER2 amplification^30^. In human breast cancer, for instance, HER2 gene amplification is not associated with the overexpression of HER2 protein^30^. CTCs are shed from primary tumor foci into the peripheral circulation or invade blood vessels through epithelial-mesenchymal transition (EMT)^31^. The isolation of CTCs from peripheral blood has emerged as a reliable alternative source of tumor cells, yielding prognostic and/or predictive biomarkers^32,33^. For example, the potential clinical utility of CTCs may contribute to the selection of HER2-positive patients with CTCs which are likely to benefit from trastuzumab treatment despite being histologically HER2-negative^34^. Another major advantage of CTC profiling is the ease of obtaining samples for monitoring tumor evolution and studying the mechanism of acquired drug resistance^35^. Genomic analyses of CTCs from patients with non–small cell lung cancer have identified the T790M gatekeeper mutation, which confers resistance to epidermal growth factor receptor (EGFR) tyrosine kinase inhibitors^35^. Furthermore, next-generation sequencing^13^ of breast cancer CTCs revealed significant inter-patient heterogeneity, which can be monitored over time^35^. Therefore, real-time assessment of tumor dynamics using CTCs is critical for selecting patients who are likely to benefit from anti-HER2 treatment.

In this study, we aimed to investigate HER2 expression in CTCs and compare it to that in tumor tissue specimens from 27 patients with metastatic gastric cancer (mGC). We used an On-chip Sort system to capture and detect CTCs undergoing EMT, using cytokeratin and vimentin as markers for epithelial and mesenchymal cells, respectively. To identify the genomic profiles underlying HER2 expression in CTCs, in real-time, DNA extracted from CTCs was used to analyze over 50 cancer-related genes using a gene panel. Thus, we aimed to elucidate the clinical significance of CTCs, rather than that of tissue specimens, by exploring their genomic profiles.

## Results

### Clinicopathological features and treatments in 27 patients

The characteristics of the 27 patients with mGC recruited for this study are shown in Table 1. Twenty-seven patients had 19 metastatic cancers, and eight had cancers with recurrence after surgery. According to the criteria for histological HER2 assessment determined using immunohistochemistry (IHC) and fluorescence in situ hybridization (FISH), 13 patients (48%) were histologically HER2-positive, whereas 14 patients (52%) were histologically HER2-negative. HER2 status, as determined by IHC and FISH, is shown in Supplementary Table S1. Ten patients had positive HER2 IHC scores of (3+), while six patients showed negative (0, 1+), and 11 patients were equivocal (2+). In these 11 patients with equivocal HER2 IHC scores, FISH was performed, which revealed that three patients were HER2 positive (threshold ratio > 2.0) and eight patients were HER2 negative. Based on the histological HER2 status, the first-line chemotherapy was determined as HER2-targeted therapy for 13 patients but not for 14 patients (no HER2-targeted therapy) (Table 1).

**Table 1:** Clinicopathological features and treatments in 27 patients with metastatic gastric cancer.

Characteristic		Value (*N* = 27)	%
Sex	Male/Female	22	81.5
	Female	5	18.5
Median age, years (range)		73 (56–80)	
ECOG PS	0	7	25.9
	1	20	74.1
Disease status	Initially metastatic	19	70.4
	Recurrent	8	29.6
Metastatic or recurrent sites	Peritoneum	13	48.1
	Liver	9	33.3
	Lymph node	12	44.4
	Others	6	22.2
Bormann type	Type 1	3	11.1
	Type 2	10	37.0
	Type 3	5	18.5
	Type 4	3	11.1
	ND	6	22.2
Histological type	Differentiated	11	40.8
	Undifferentiated	16	59.2
HER2 status	Positive	13	48.1
	Negative	14	51.9
HER2-targeted therapy	Present	13	48.1
	Absent	14	51.9

ECOG PS, Eastern Cooperative Oncology Group performance status; HER2, human epidermal growth factor receptor 2; IHC, immunohistochemistry; FISH, fluorescence in situ hybridization; ND, not determined.

## Detection of CTCs to investigate HER2 expression and degree of EMT in CTCs

**Fig. 1:**
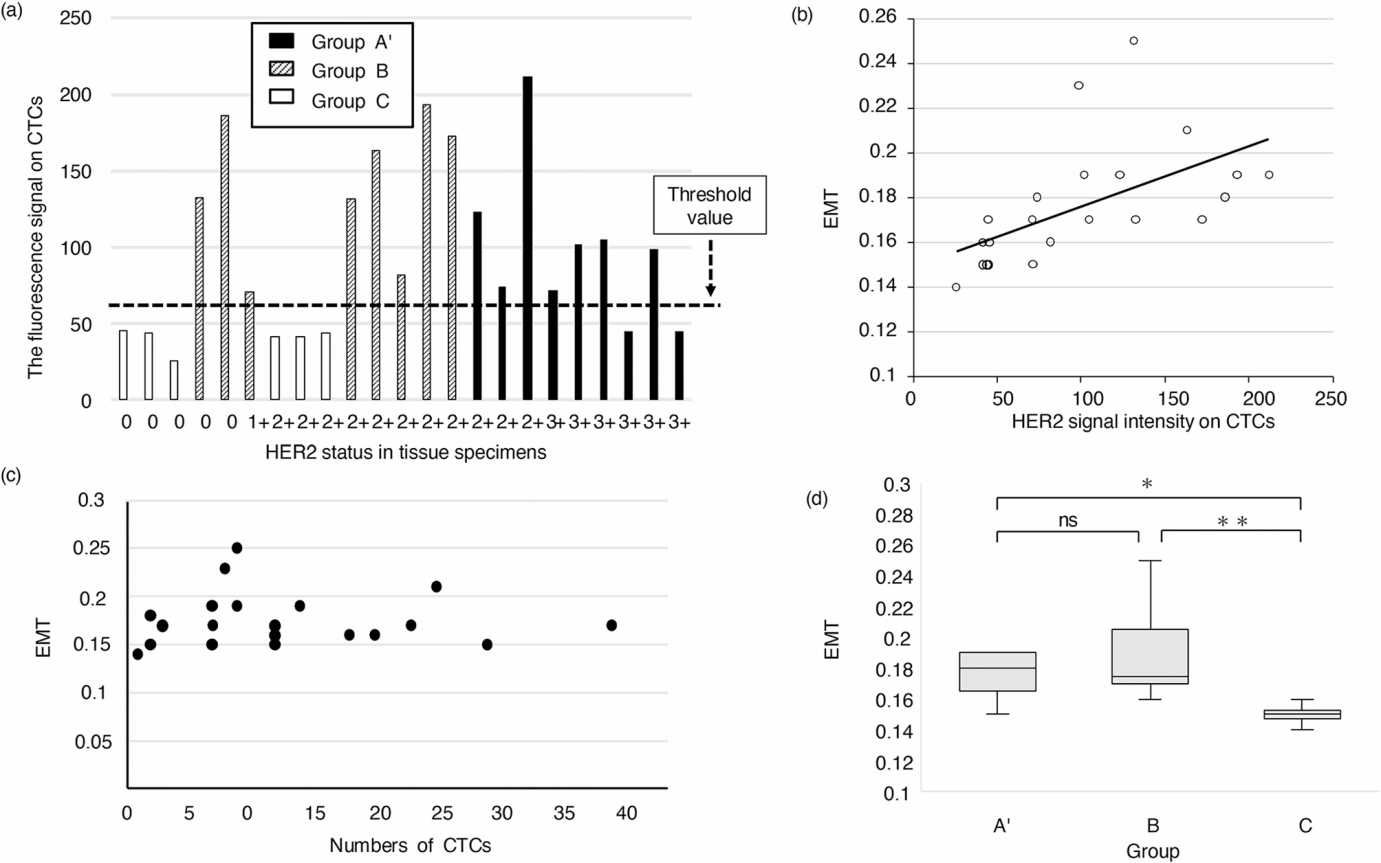
**HER2 expression in CTCs and tumor tissue specimens related to epithelial-mesenchymal transition (EMT) in 23 patients with gastric cancer** (**a**) HER2 expression in CTCs and tumor tissue specimens. The status of histological HER2 expression was investigated by immunohistochemistry (IHC) and/or fluorescence in situ hybridization (FISH) in tumor tissues resected or biopsied from patients. HER2 status in CTCs was assessed using fluorescence signal intensity in CTC membranes and measured using an On-chip Sort system (On-chip Biotechnologies). The signal intensity of HER2 fluorescence > 56 was defined as positive expression of HER2 in CTCs. The threshold value of HER2 expression in CTCs identified three groups, patients with HER2 expression in tumors (group A’, which included 9 patients with CTC detection who were assigned to group A), patients without HER2 expression in tumors but harboring HER2 expression in CTCs (group B), and patients without HER2 expression in tumors or CTCs (group C). (**b**) Significant correlation between EMT-index and HER2 expression in CTCs (*p* < 0.001, Spearman’s correlation coefficient = 0.765). The EMT-index was calculated from the ratio of the fluorescence signal intensity of cytokeratin and vimentin: fluorescence signal intensity of vimentin/(fluorescence signal intensity of cytokeratin + fluorescence signal intensity of vimentin). (**c**) No significant correlation between the number of CTCs and the EMT-index (*P* = 0.693). (**d**) Comparison of EMT-index between the three groups. ns; no significance, ^*^P = 0.05, ^**^P = 0.005. CTCs, circulating tumor cells.

CTC detection was conducted to investigate HER2 expression in 23 of the 27 patients recruited for this study, comprising 14 patients without HER2 expression and nine patients with HER2 expression in their tumor tissues. CTCs were detected in all 23 patients. The median CTC count was nine cells (range: 1–39 cells; Table 2). Based on the signal intensity of HER2 on CTCs, which ranged from 26 to 212, 15 patients were positive for HER2 expression in CTCs (threshold value > 56), and eight were negative. The 15 patients with HER2-positive CTCs included eight patients without HER2 expression in their tumor specimens. Six patients showed HER2-negative in both CTCs and tumor tissues while 7 patients exhibited HER2-positive in both CTCs and tumor tissues. Consequently, our data showed a 57% concordance (13 of 23 patients) between HER2 expression in tumor tissues and CTCs (Table 2). Figure 1a shows the relationship between HER2 expression in CTCs and tumor tissues. The threshold value of HER2 expression in CTCs (threshold value > 56) identified three groups: patients with HER2 expression in tumors (group A’, which included 9 patients with CTC detection who were assigned to group A), patients without HER2 expression in tumors, but with HER2 expression in CTCs (group B), and patients without HER2 expression in tumors or CTCs (group C).

**Table 2:** Detection of CTCs and HER2 status as well as EMT-index in 23 patients with metastatic gastric cancer.

Case	Number of CTCs	HER2 signal intensity on CTCs	HER2 status on CTCs	EMT-index	Histological HER2 status
1	2	45	Negative	0.15	Negative
2	12	44	Negative	0.15	Negative
3	1	26	Negative	0.14	Negative
4	39	133	Positive	0.17	Negative
5	2	186	Positive	0.18	Negative
6	7	71	Positive	0.17	Negative
7	29	41	Negative	0.15	Negative
8	20	41	Negative	0.16	Negative
9	7	44	Negative	0.15	Negative
10	9	131	Positive	0.25	Negative
11	25	163	Positive	0.21	Negative
12	12	82	Positive	0.16	Negative
13	7	193	Positive	0.19	Negative
14	3	172	Positive	0.17	Negative
15	14	123	Positive	0.19	Positive
16	2	74	Positive	0.18	Positive
17	7	212	Positive	0.19	Positive
18	7	72	Positive	0.15	Positive
19	9	102	Positive	0.19	Positive
20	12	105	Positive	0.17	Positive
21	23	45	Negative	0.17	Positive
22	8	99	Positive	0.23	Positive
23	18	45	Negative	0.16	Positive

HER2, human epidermal growth factor receptor 2; IHC, immunohistochemistry; CTCs, circulating tumor cells; EMT, epithelial–mesenchymal transition. In the CTC analysis, only 23 patients were included while 27 were recruited overall.

## Degree of EMT and its association with HER2 expression in CTCs

We then elucidated the degree of EMT in relation to HER2 expression in the CTCs of the 23 patients (Table 2). The median EMT-index was 0.17 (range: 0.15–0.24). There was a significant correlation between the EMT-index and HER2 expression in CTCs (p < 0.001, Spearman’s correlation coefficient = 0.765; Fig. 1b). On the other hand, there was no significant correlation between the number of CTC and the EMT-index (P = 0.693, Fig. 1c), which indicated that CTCs could be detected regardless of EMT progression. In general, EMT progression makes it difficult to detect CTCs when using epithelial cell adhesion molecule (EpCAM); however, the On-chip Sort system overcame the risk of missing CTCs with EMT progression. We then compared the EMT-index among the three groups within the 23 patients. The median EMT-index in groups A’, B, and C were 0.18 (0.14–0.23), 0.17 (0.16–0.25), and 0.15 (0.14–0.16), respectively. The median EMT-index of groups A’ and B were significantly higher than those of group C (*p = 0.05 and **p = 0.005, respectively, Fig. 1d). There were no significant differences in the median EMT-index between groups A’ and B (Fig. 1d).

## Comparison of clinicopathological features between the three groups

**Fig. 2:**
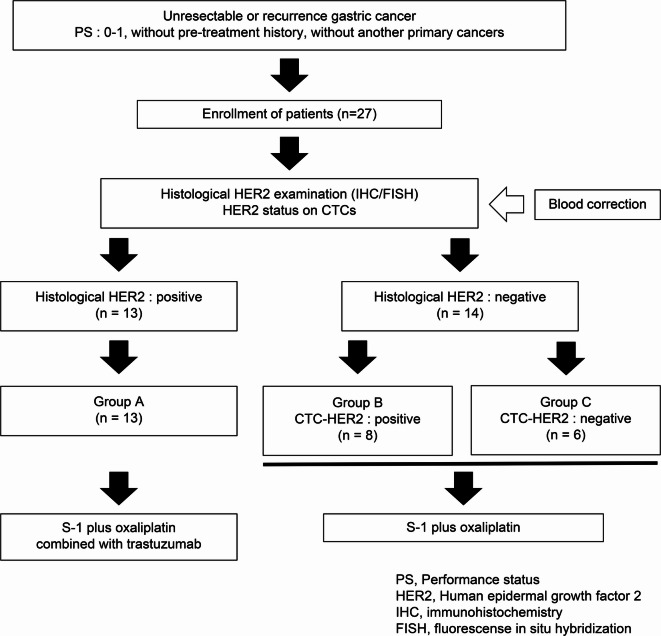
**Schematic presentation of the study design** Peripheral blood samples were collected from 27 patients with GC, and the presence or absence of CTCs was assessed using the On-chip Sort system. HER2 expression in tumor tissues and CTCs was investigated. Based on this status, the patients were classified into three groups: HER2-positive tumor tissues (group A), HER2-negative tumor tissues but HER2-positive CTCs (group B), and HER2-negative tumor tissues and CTCs (group C). In group A, patients received S-1 plus oxaliplatin (SOX) combined with trastuzumab. The patients in groups B and C received SOX. Patients received the study treatment until disease progression, unacceptable toxicity, or withdrawal of consent.

Based on the HER2 status of the tumors and CTCs, the 27 patients were divided into three groups. Thirteen patients with HER2 expression in tumors were classified as group A, eight patients without HER2 expression in tumors but with HER2 expression in CTCs were classified as group B, and six patients without HER2 expression in tumors or CTCs were classified as group C (Fig. 2). Thirteen patients with HER2 positive expression in tumors (group A) were treated with S-1 plus oxaliplatin combined with trastuzumab, a HER2-targeted therapy, while 14 patients without HER2 positive expression in tumors (group B or C) were treated with S-1 plus oxaliplatin but no additional trastuzumab. Clinicopathological features were compared among the three groups. There were significant differences in the clinicopathological features between these three groups in disease status, anastomotic/recurrent sites, histology, and HER2 status (Table 3). In addition, we adjusted this comparison by evaluating 23 patients with CTC detection including 9 patients with HER2 expression in tumors (group A’); 8 without HER2 expression in tumors, but with HER2 expression in CTCs (group B); and 6 without HER2 expression in tumors or CTCs (group C). Sex, disease status, metastatic or recurrent sites (anastomotic/remnant and peritoneum), and histology were significantly different between the three groups (Supplementary Table S2).

**Table 3:** Comparison of clinicopathological features between the three groups in 27 patients with metastatic gastric cancer, based on HER2 status in tumors and CTCs.

Total (*n* = 27)	Group A *n* = 13 (%)	Group B *n* = 8 (%)	Group C *n* = 6 (%)	*P* value
Sex, male	11 (84.6)	8 (100.0)	3 (50)	0.054
Age, > 71	7 (53.8)	4 (50)	3 (50)	0.980
PS, 1	10 (76.9)	6 (75)	4 (66.7)	0.891
Disease status				
Initially metastatic	11 (84.6)	3 (37.5)	6 (100)	0.015
Recurrent	2 (16.7)	5 (62.5)	0 (0)	0.015
Metastatic or recurrent sites				
Anastomotic/Remnant	0 (0)	3 (37.5)	0 (0)	0.018
Peritoneum	8 (61.5)	3 (37.5)	2 (33.3)	0.402
Liver	5 (38.5)	2 (25)	2 (33.3)	0.817
Distant lymph nodes	4 (30.8)	3 (37.5)	5 (83.3)	0.090
Other sites	2 (15.4)	1 (12.5)	0 (0)	0.605
Bormann, type 4	0 (0)	1 (12.5)	2 (33.3)	0.113
Histology, Undifferentiated	4 (30.8)	7 (87.5)	5 (83.3)	0.015
HER2 status, positive	13 (100)	0 (0)	0 (0)	< 0.001

Data are presented as n (%). HER2, human epidermal growth factor receptor 2.

## Comparison of progression free survival between the three groups

**Fig. 3:**
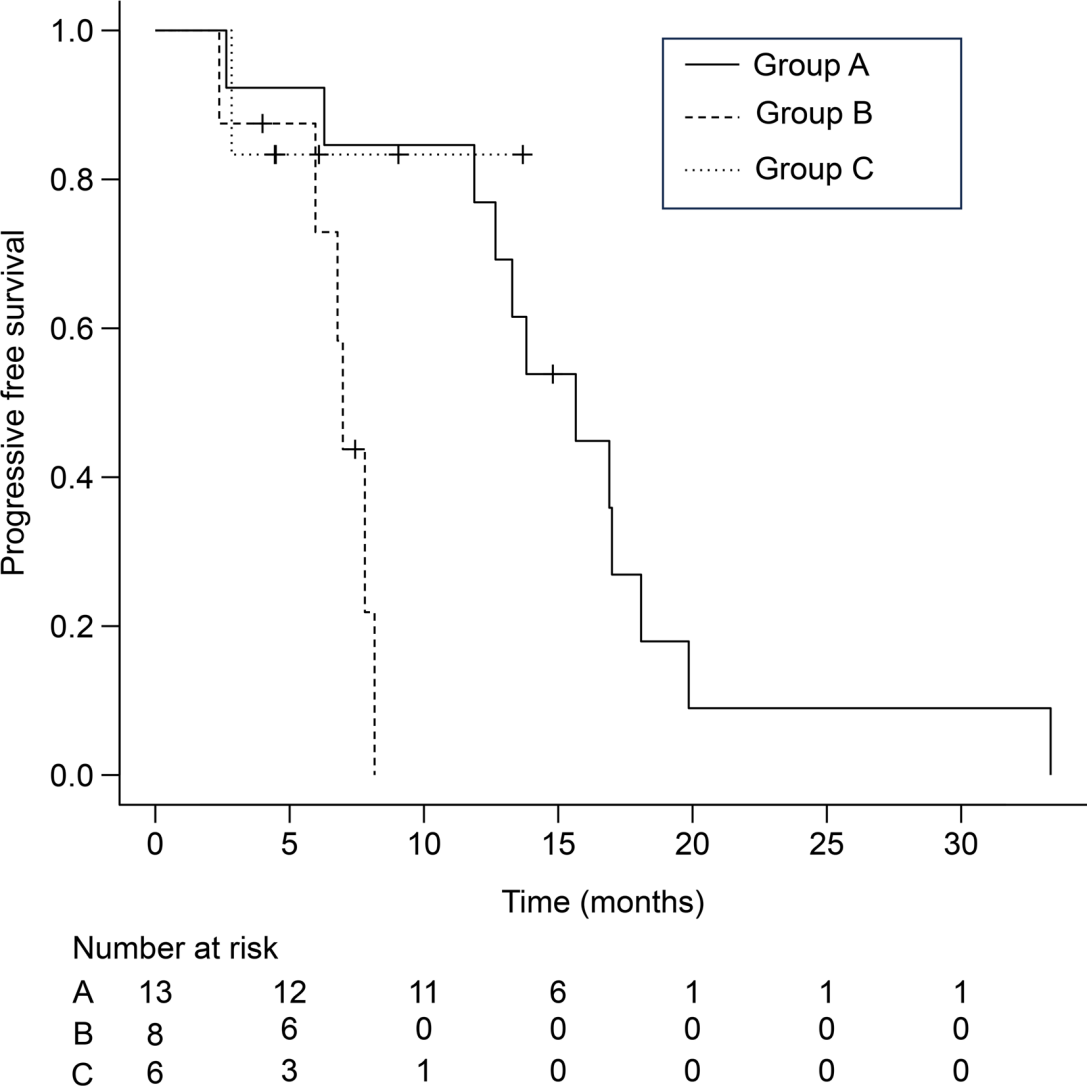
Comparison of progression-free survival between the three groups. The median PFS for groups A, B, and C was 15.7 months (95% CI, 11.9 to 18.1), 7.0 months (95% CI, 2.38 to NA), and not reached (95% CI, 2.84 to NA), respectively (*p* = 0.012).

Progression-free survival (PFS) was compared among the three groups based on HER2 status in tumors and CTCs. There was a significant difference in PFS between the three groups (15.7 months in A, 7.0 months in B, and not reached in C, *p* = 0.012, Fig. 3). Group B exhibited worse PFS than the other groups, suggesting that patients with HER2 expression in CTCs had a worse prognosis without trastuzumab treatment. Considering the HER2 expression in the CTCs of these patients, they may be potential candidates for trastuzumab indication, regardless of histological HER2 status. Additionally, we adjusted this comparison by evaluating 23 patients with CTC detection. There was a significant difference in PFS between the three groups (16.9 months in group A’, 7.0 months in B, and not reached in C, *p* = 0.008, Supplementary Figure S1). Group B exhibited worse PFS than the other groups.

### Comparison of genomic profiles between the three groups

**Fig. 4:**
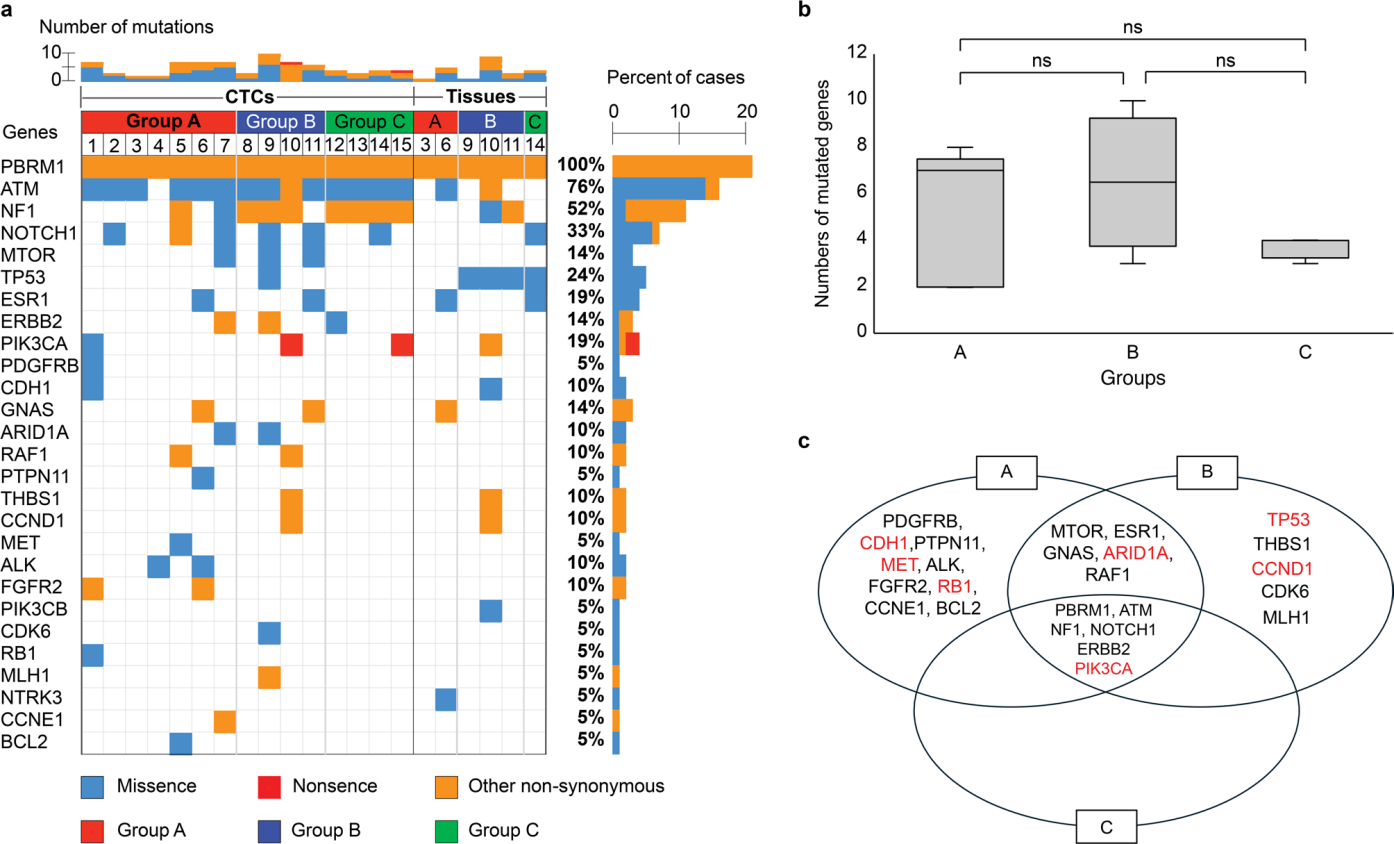
**The landscape of genomic alterations compared between the three groups** (**a**) The heat map on the left shows the genomic change landscape of the CTC samples, and on the right is that of the tissue samples. Number of cases (%) is shown next to the heat map. Genomic alterations were compared between the three groups, A, B and C, respectively. Patients are ordered by case number and identical numbers indicate samples from identical patients. The colors below indicate the specific types of genomic alterations including missense mutations (blue), nonsense mutations (red), other non-synonymous mutations (orange). Each column indicates a sample and each row represents a gene. The landscape includes 27 altered genes among 53 genes. (**b**) Comparison of numbers of mutated genes between the three groups. The median numbers of mutated genes per case were 7.0 (2–8), 6.5 (3–10), and 4.0 (3–4) in groups A, B, and C, respectively. ns; no significance. (**c**) Venn diagrams of mutated genes compared between the three groups. EMT-related genes are shown in red.

**Fig. 5:**
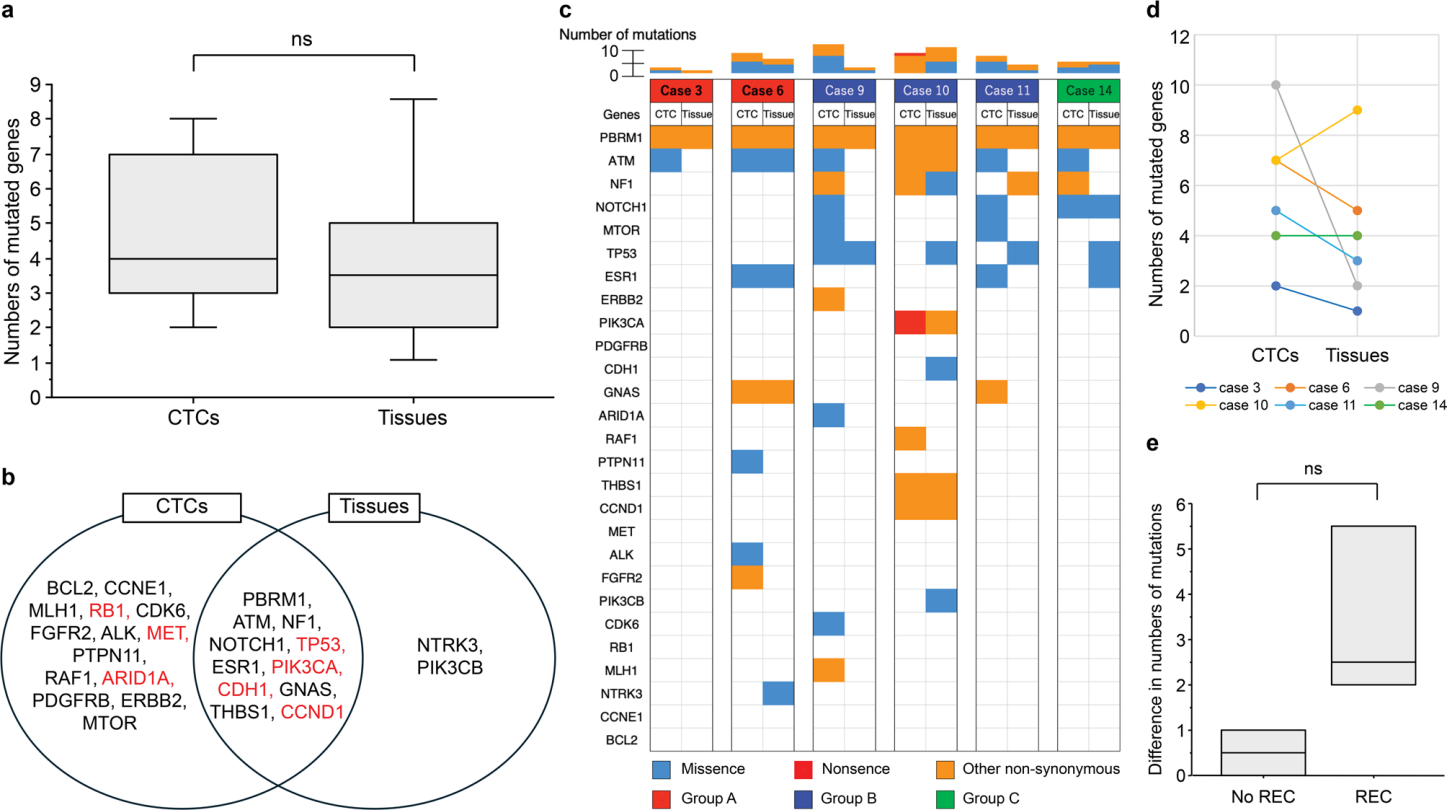
**Comparison of genomic alterations between CTCs and tumor tissues** (**a**) Comparison of numbers of mutated genes between CTCs and tumor tissues. The median number of gene mutations in CTCs and tissues was 4.0 (2–10) and 3.5 (1–9), respectively. ns; no significance. (**b**) Venn diagram of mutated genes between CTCs and tumor tissues. EMT-related genes are shown in red. (**c**) The landscape of genomic alterations compared between CTCs and tumor tissues in six identical individuals. The colors indicate different groups. (**d**) Comparison of numbers of mutated genes between CTCs and tissues in six identical individuals. (**e**) Comparison of numbers of genome mutations between patients with GC recurrence (REC, *n* = 4) and those without recurrence (No REC, *n* = 2).

To identify the genomic profiles underlying HER2 expression in CTCs, genomic analysis of 53 cancer-related genes was performed. Among the 27 patients examined, tumor tissue DNA was obtained from six patients, and CTC DNA was obtained from 15 patients to confirm the occurrence of somatic mutations. The samples were subjected to genomic analysis after whole-genome amplification (WGA). WGA can introduce technical artifacts, such as loss of coverage and reduced uniformity, which may hinder the detection of rare genetic mutations. Among the various WGA methods available, we utilized the Ampli1 WGA kit, which has been reported to offer superior performance in terms of coverage and reproducibility^36^. When comparing the average depth of coverage for each gene between CTC and tissue samples, the coverage was lower in CTC samples than in tissue samples. However, 96% of genes achieved a coverage depth of at least 100x in CTC samples (Supplementary Figure S2). These findings indicate that the Ampli1 WGA kit maintains sufficient coverage to reliably detect gene mutations in CTC samples. We found gene mutations in CTCs, such as *APC*, *MET*, *CDH1*, *ERBB2*, *FGFR2*, *PIK3CA*, and *TP53*. These gene mutations are frequently observed in tissue specimens of tumors with chromosomal instability and are classified as one of the molecular subtypes of GC^7^. A subgroup of tumors expressing HER2 was included in this molecular subtype. Figure 4a shows the landscape of genomic alterations using CTCs and tumor tissues compared among the three groups. Twenty-seven mutated genes were identified and listed in this landscape. The landscape of all 53 genes is shown in Supplementary Figure S3. The concordance of genomic profiles between the three groups was not high, but a high number of mutations was observed in both groups A and B compared to group C, although the difference was not significant. The median numbers of gene mutations in groups A, B, and C were 7.0 (2–8), 6.5 (3–10), and 4.0 (3–4), respectively (Fig. 4b). A Venn diagram of gene mutations in CTCs identified six mutations–*PBRM1*,* ATM*,* NF1*,* NOTCH1*,* ERBB2*, and *PIK3CA*–that were shared among the three groups (Fig. 4c). *PIK3CA* is involved in the mechanisms underlying EMT^37^. *MTOR*,* ESR1*,* GNAS*,* ARID1A*, and *RAF1* were shared between groups A and B. *ARID1A* is involved in mechanisms underlying EMT^38^. *PDGFRB*,* CDH1*,* PTPN11*,* MET*,* ALK*,* FGFR2*,* RB1*,* CCNE1*, and *BCL2* were only detected in group A, and *TP53*,* THBS1*,* CCND1*,* CDK6*, and *MLH1* were only detected in group B. Group C had no mutations, except for the six gene mutations shared between the three groups. *CDH1*,* MET*,* RB1*,* TP53*, and *CCND1*, detected in groups A or B, are involved in the mechanisms underlying EMT^39–41^. Many gene mutations involved in the mechanisms underlying EMT were found in groups A or B, but only one gene was found in group C, indicating that group B harbored similar oncogenic features to group A with regard to the EMT induction. A comparison of the mutated genes between CTCs and tumor tissues is shown in Fig. 5a and **b**. CTCs were likely to show greater number of gene mutations compared to tumor tissues, but there was no significant difference in these number (Fig. 5a). The median numbers of gene mutations in CTCs and tissues were 4.0 (2–10) and 3.5 (1–9), respectively. A Venn diagram of genomic alterations demonstrated that 11 gene mutations, including *PBRM1*,* ATM*,* NF1*,* NOTCH1*,* TP53*,* ESR1*,* PIK3CA*,* CDH1*,* GNAS*,* THBS1* and *CCND1* were shared between CTCs and tissue specimens (Fig. 5b). An additional 14 and two mutated genes were identified in the CTCs and tissues, respectively. *RB1*,* MET*, and *ARID1A* detected in CTCs, and *TP53*,* PIK3CA*,* CDH1*, and *CCND1* detected in both CTCs and tissues are involved in the mechanisms underlying EMT^39–41^. Figure 5c shows the landscape of gene mutations when comparing CTCs and tumor tissues in six identical individuals. CTCs were likely to have more genetic mutations than tumor tissues (Fig. 5d). We then compared the difference in the number of gene mutations between CTCs and tumor tissues according to disease status, which revealed that patients with recurrence (REC) displayed a greater number of gene mutations than those without recurrence (No REC), although the difference was not significant. The median number of gene mutations in patients with and without recurrence, were 2.5 (2–8) and 0.5 (0–1), respectively (Fig. 5e).

## Discussion

In this study, we investigated HER2 expression in CTCs and compared it to that in tumor tissue specimens from 27 patients with mGC. The EMT-index of CTCs was also assessed and was found to be significantly associated with HER2 expression. An increased EMT-index was observed in patients with HER2 expression, regardless of the source being CTCs or tissue specimens, indicating that patients with HER2 expression are likely to have metastasis. Based on investigation of the HER2 status of tumors and CTCs, we identified 13 patients with HER2 expression in tumor tissues (group A), eight patients with HER2 expression in CTCs despite no HER2 expression in tumor tissues (group B), and six patients without HER2 expression in tumors or CTCs (group C). Group B exhibited worse PFS than the others (13.8 months in A, 7.0 months in B, and not reached in C, *p* = 0.012, Fig. 3). We adjusted the PFS comparison by evaluating 23 patients with CTC detection. The adjustment showed consistent results. In addition, genomic analysis identified cancer gene mutations, some of which are involved in the mechanisms underlying EMT. Groups A and B likely harbored several EMT-related gene mutations. Considering the oncological features of group B, an additional, HER2-targeted therapy may be beneficial. Our study suggests that real-time assessment of CTCs can refine the indication for HER2-targeted therapies in patients with metastatic GC.

EpCAM expressed on the cell surface has been used as a marker of epithelial cells in the blood to detect CTCs. However, for patients with GC, EpCAM carries the risk of missing the most aggressive CTC subpopulations because of EMT, potentially leading to an underestimation of the total number of CTCs present in the bloodstream^35^. Some CTCs do not express EpCAM on their surface^28,42,43^. In the present study, the On-chip Sort system was used to detect CTCs expressing cytokeratin but not EpCAM, such as cells derived from tumors undergoing EMT^44^. We identified CTCs in all 23 patients with GC, using the On-chip Sort system. Matsushita reported that CTCs were detected in 62% of patients with GC, based on EpCAM expression^35^. In our study, CTCs were detected in 100% of patients, based on cytokeratin expression.

The discordance in HER2 expression or amplification between tissue and blood samples is a critical issue in the clinical application of liquid biopsies. Tumor tissue specimens are the main source for evaluating HER2 status in patients with GC^17–19^,however, a positive HER2 status can be misdiagnosed as negative in patients with GC, when the diagnosis is based on tissue biopsy; this is due to tumor heterogeneity^18,28^. Shitara et al. reported a 64% concordance between HER2 expression in tumor tissues and HER2 (ERBB2) plasma gene amplification using circulating free DNA (cfDNA)^45^. Matsushita et al. reported 54% concordance between HER2 expression in tumor tissues and HER2 (ERBB2) expression in CTCs^34^, which is consistent with our data of 57% concordance. Identification of HER2 expression or amplification by liquid biopsy demonstrated the clinical benefits of trastuzumab treatment in patients with GC whose CTCs or cfDNA showed HER2 expression or amplification, respectively, although their primary tumors were histologically HER2 negative^34,45^. Liquid biopsy is increasingly recognized as a more appropriate tool for the evaluation of tumor genetic features of HER2 than tissue specimens in patients with GC^5,28^.

EMT is a pivotal step in cancer progression, in which cells acquire motility by losing epithelial characteristics and gaining mesenchymal features^46^. The malignant progression of many types of carcinomas, quite possibly all of them, depends on EMT activation in neoplastic cells^47–49^. EMT causes cancer cells to lose their adhesive and proliferative properties, enabling them to invade blood vessels—a critical step closely associated with metastasis in various cancers, including breast, pancreatic, lung, colorectal, hepatocellular, and bladder cancers^50^. Additionally, HER2 overexpression has been shown to induce EMT and promote metastasis in D492 breast epithelial progenitor cells^51^. In gastric cancer cell lines such as SNU-216 and NCI-N87, HER2 promotes metastasis via the AKT/JNK/EMT signaling pathway^52^. Our study demonstrates that the EMT-index is significantly associated with HER2 expression. EMT is a complex biological process that cannot be fully defined by specific markers alone. A comprehensive evaluation of EMT requires protein expression analysis and the consideration of cellular characteristics. Qualitative assessment of EMT induction typically involves measuring a decrease in epithelial markers, such as cytokeratin, alongside an increase in mesenchymal markers, such as vimentin^51,52^. In addition, several transcription factors belonging to the Snail, Twist, and Zeb families have been found to control cell–cell adhesion, cell migration, and extracellular matrix degradation, and to play evolutionarily conserved central roles in the execution of EMT in various biological settings and organisms^53^. In this study, EMT induction was assessed using the EMT-index, which quantitatively integrates the contrast between cytokeratin and vimentin expression. An increased EMT-index was observed in patients with HER2 expression, regardless of the source being CTCs or tissue specimens, indicating that patients with HER2 expression are likely to have metastasis. In addition, our study identified eight patients without HER2 expression in tumor tissues but expressing HER2 in CTCs, showing a worse prognosis when they were treated with cytotoxic agents without combined HER2-targeted therapy. In contrast, eight patients with HER2 expression in tumor tissues displayed a better prognosis when they were treated with cytotoxic agents and HER2-targeted therapy. These data suggest that patients with HER2 expression in CTCs, but not in tumor tissues, had a poor prognosis without HER2-targeted therapy. Considering HER2 expression in CTCs in these eight patients, additional HER2-targeted therapy may be beneficial for these patients. The benefit of HER2-targeted therapy was reported in patients with HER2 expression in CTCs rather than in patients with HER2 expression in tumor tissues^34,54^, indicating that HER2 status on CTCs may be a promising blood marker for HER2-targeted therapy compared to that of tumor tissues. In breast cancer patients, HER2-targeting therapies, such as HER2-antibody drug conjugates (ADCs), was reported to be effective for patients with HER2 low tumors measured using tissue specimens. However, there is no evidence of the introduction of HER2-ADCs for mGC patients with HER2 low tumors. This study on CTC-based liquid biopsy provides important insights into the clinical implications of HER2-targeting therapies including HER2-ADCs for patients with HER2-low metastatic gastric cancer, as assessed from tissue specimens. In addition, Park et al. reported that patients with rescued HER2 positivity can be offered the same benefits of trastuzumab-containing chemotherapy as those showing HER2 positivity at initial assessment^18^. Conversely, our study identified two patients with histologically HER2-positive tumors who were HER2-negative in their CTCs. HER2 expression in tumor tissues for these patients was evaluated using biopsy specimens. It is speculated that the small portion of tumor tissue obtained through biopsy may not accurately represent the tumor’s overall characteristics. Intratumoral heterogeneity, where HER2-negative cells constitute a significant proportion of the tumor, may account for the discrepancy. Consistent with our findings, Matsushita et al. reported that 11 out of 27 cases with HER2-positive tissues exhibited HER2-negative CTCs^34,54^, underscoring the potential misalignment between CTC HER2 status and tissue HER2 status due to biological differences or sampling limitations. Furthermore, HER2 inhibitors may have limited efficacy in patients without HER2 expression in CTCs despite HER2 positivity in tumor tissues. However, the detection of HER2 positivity in CTCs remains critically important, as it offers a valuable avenue for refining treatment strategies and improving clinical outcomes. These findings emphasize the potential of CTC-based HER2 assessment in enhancing the precision of HER2-targeted therapies. In this study, HER2 overexpression in CTCs was defined as mean signal intensity of HER2 fluorescence > 56 in the CTC membranes. On the other hand, considering the limited number of CTCs, the number of HER2 positive CTCs among total CTCs could account for HER2 status in CTCs involved in drug sensitivity.

Genomic analysis of CTCs was performed to investigate the genomic profiles underlying tumors associated with HER2 expression in CTCs. Although genomic analysis of tumor tissues has been performed previously, in patients with GC^1,7,8^., no study has been conducted using next-generation sequencing of CTCs in patients with mGC. Because the number of CTCs isolated from blood is small, the amount of DNA is insufficient for direct NGS analysis. Therefore, whole-genome amplification is necessary and consequently, contamination-free purification of CTCs is important. In the present study, an On-chip Sort system was used for the purification of CTCs. This system performs sorting by using a disposable microfluidic chip. The chip included a reservoir each for the sample, sheath fluid, collection, and waste. The use of different chips to process CTCs from different patients prevented cross-contamination between samples. Our study identified several gene mutations in CTCs that are frequently observed in tumors with chromosomal instability (CIN) and are classified as one of the molecular subtypes of GC^7^. This molecular subtype includes a subgroup of tumors that express HER2. The identification of gene mutations in CTCs, frequently occurring in specific types of tumors, has enabled the monitoring of tumor dynamics involved in temporal, spatial, and intertumoral heterogeneities. In this study, the landscape of genomic alterations in CTCs exhibited genomic profiles in all three groups. We expected to observe similar genomic profiles amongst patients with HER2 expression, regardless of the source being CTCs or tumor tissues; however, the concordance was not high. This may be because Oncomine Tumor Mutation Load can detect single nucleotide variants (SNVs), and insertions and deletions (INDELs). Expanding investigations to target other genetic alterations, including copy number variations (CNVs), structural rearrangements, and gene fusions, may provide different views of the landscape. In contrast, a high number of mutations were observed in both groups A and B compared to group C, although the difference was not significant (Fig. 4b). In addition, Venn diagrams of genomic alterations displayed gene mutations shared between the groups, some of which were involved in the mechanisms underlying EMT (Fig. 4c). These EMT-related genes were likely observed in groups A and B, but not in group C, indicating that group B harbored similar oncogenic features to group A with regard to EMT induction. Group B may be offered the benefits of HER2-targeted therapy, similar to group A. Real-time assessment of CTCs can therefore refine the indication for HER2-targeted therapies in patients with metastatic GC. Comparing the numbers of gene mutations between CTCs and tissue specimens from six identical individuals, case 9 showed a large difference in the number of gene mutations, such as 10 gene numbers in CTSs and 2 gene mutations in tissue specimens. Case 9 involved a patient with recurrence. Tissue specimens for genomic analysis were obtained from patients with GC during the primary operation, which was performed three years ago. This time lag and the different sample sources may result in temporal, spatial, and intertumoral heterogeneities. Our study showed that patients with recurrence displayed greater number of genetic mutations than those without recurrence, although the difference was not significant. Overall, liquid biopsy using CTCs is a promising tool for providing real-time information on tumor features.

The present study has some limitations. This retrospective cohort study was conducted at a single institution with a relatively small number of enrolled patients, which may limit the generalizability of the findings. Additionally, the analysis of CTC in this study was conducted as a one-time evaluation without longitudinal monitoring. For effective monitoring, sequential sampling over time is necessary to assess CTC dynamics and their potential utility as a real-time biomarker. While the current approach provides limited value for monitoring treatment response, it may be more appropriate for stratifying patients based on baseline characteristics. As EMT exists on a spectrum, a comprehensive assessment would require multiple markers. Owing to channel limitations in the On-chip Sort system, we employed only vimentin as the mesenchymal marker in this study. CTCs with reduced cytokeratin signals due to further EMT progression are considered undetectable. Genomic analysis using Oncomine Tumor Mutation Load can only detect gene mutations including SNVs and INDELs. Patients with HER2 expression in CTCs are expected to benefit from additional HER2-targeted therapy; however, no data were obtained upon their treatment with HER2-targeted therapy. Further studies are required to explore the potential of CTC-based therapeutic interventions for mGC. In addition, the selection of chemotherapy regimens based on CTC monitoring appears feasible yet requires extensive clinical validation in interventional trials.

In summary, the detection of HER2 expression in CTCs identified patients who needed therapeutic interventions, specifically with indications for HER2-targeted therapy. The real-time assessment of genomic profiles using CTCs may lead to a paradigm shift in cancer treatment strategies. Although our findings should be interpreted within the study limitations, and further examinations may be required to draw definitive conclusions, we believe that our study provides important insights into the appropriate selection of treatment strategies for patients with mGC.

## Methods

### Cell lines and culture

MKN7, GSU, NUGC4 (RIKEN BRC, Ibaraki, JAPAN, HER2-positive gastric cell lines), and KATOIII (RIKEN BRC, Ibaraki, JAPAN, a HER2-negative gastric cell line) cells were cultured in Roswell Park Memorial Institute (RPMI) 1640 (Sigma-Aldrich, St. Louis, MO, USA) supplemented with 10% fetal bovine serum (HyClone Laboratories, Logan, UT, USA). All cell lines were incubated at 37 ºC in a humidified atmosphere containing 5% CO_2_. These cell lines were used as controls to determine the threshold value for HER2-positive CTCs.

### Patients and study design

We prospectively recruited 27 patients with unresectable or recurrent GC and performed first-line chemotherapy between October 2020 and October 2021. We collected blood samples prior to or during early chemotherapy, from the same patients at Saitama Medical Center, Jichi Medical University, Japan. This was an exploratory study that did not calculate the sample size for the primary endpoints. All patients had unresectable or recurrent adenocarcinoma of the stomach confirmed by pathological and imaging examinations, such as esophagogastroduodenoscopy, computed tomography, and/or fluorodeoxyglucose positron emission tomography. The eligibility criteria included an Eastern Cooperative Oncology Group performance status (PS) score of 0 or 1 and a life expectancy > 3 months. Exclusion criteria were second malignancy, chronic heart failure with less than 50% left ventricular ejection fraction (LVEF), or uncontrolled comorbid medical conditions. Histological HER2 expression was investigated by IHC and/or FISH in tumor tissues resected or biopsied from these patients. Additionally, peripheral blood samples were collected, and CTCs were detected using the On-chip Sort system. Furthermore, HER2 expression in CTCs was analyzed using the On-chip Sort system. Finally, the patients were classified into three groups: histologically HER2-positive (group A), histologically HER2-negative and CTC HER2-positive (group B), and histologically HER2-negative and CTC HER2-negative (group C; Fig. 2). In group A, patients received cytotoxic agents in combination with trastuzumab. In groups B and C, patients received cytotoxic agents only. PFS was defined as the time from the date of treatment initiation to the date of disease progression, death, or the last follow-up. After the diagnosis of relapsed or refractory disease, the treatment strategy was dependent on the institutional guidelines. Clinical responses were evaluated at a median of 3 months after the initial induction of first-line chemotherapy. Tumor responses were assessed using the Response Evaluation Criteria in Solid Tumors (RECIST) and classified as partial response (PR) or non-PR. All patients provided written informed consent for the examination of their tissues and plasma and use of their clinical data. The study protocol was approved by the Research Ethics Committee of Jichi Medical University, Saitama, Japan (approval no. 22–086), and conformed to the ethical guidelines of the World Medical Association Declaration of Helsinki.

### Analysis of HER2 status in tissues

HER2 status in tissues was determined by IHC analysis, which was performed on formalin-fixed, paraffin-embedded tissues using Pathway (monoclonal antibody, 4B5; Ventana Medical System, Tucson, AZ, USA) with an automated staining device, according to the manufacturer’s US Food and Drug Administration-approved procedures. HER2 protein expression was scored on a scale of 0 to 3 according to the GC consensus panel recommendations. Following the American Society of Clinical Oncologists/College of American Pathologists guidelines, IHC 0 and IHC 1 + were considered HER2-negative, whereas IHC 3 + was considered as HER2-positive. IHC 2 + was considered equivocal; a case was HER2-positive if it was IHC 2 + plus FISH with a threshold ratio > 2.0 between the HER2 gene CN and the chromosome 17 centromere (HER2:CEP17). FISH was performed only in IHC 2 + cases using the Abbott PathVysion HER2 DNA Probe Kit protocol (Abbott Laboratories, Abbott Park, Des Plaines, IL) according to the manufacturer’s instructions.

### Detection of CTCs

CTCs were detected in 23 of the 27 patients recruited for this study. Blood samples were not collected from four patients with histologically HER2-positive tumors, as they had already initiated HER2-targeted therapy before sample collection. Peripheral blood samples (8 mL) were drawn into Cell-Free DNA BCT tubes (Streck, La Vista, NE, USA); after the first 3 mL or more, blood samples were discarded to avoid epithelial cell contamination, maintained at room temperature, and processed for CTC analysis within 72 h of collection. Whole blood samples were mixed with RBC Lysis Buffer (420302, BioLegend, San Diego, USA) at a 1:10 dilution, gently pipetted, and incubated at room temperature. Following incubation, samples were centrifuged at 500 × g at room temperature. After centrifugation, the supernatant was carefully removed, and the cell pellet was washed twice by resuspending in 5 mL of T-buffer (On-chip Biotechnologies, Tokyo, Japan) containing 0.5% bovine serum albumin (BSA) (Sigma-Aldrich). The suspension was centrifuged at 500 × g for 5 min at room temperature, and the supernatant was carefully removed. The cell pellet was suspended in 1 mL of 1× working solution of Fix Concentrate (424401, BioLegend) for fixation and incubated at room temperature. Then, 1 mL of 1× working solution of Perm Buffer (424401, BioLegend) was added for permeabilization, mixed gently by pipetting and incubated at room temperature. Following incubation, samples were washed twice. For immunostaining, 1 mL of Blocker Casein in PBS (37582, Thermo Fisher Scientific) was added, mixed gently by pipetting, and incubated at room temperature. After incubation, samples were washed. The cell pellets were resuspended in 200 µL of staining solution, which contained 25 µL of Human TruStain FcX (422302, BioLegend) and a fluorescein isothiocyanate (FITC)-conjugated anti-cytokeratin antibody (CK3-6H5), which recognizes CK7, 8, 18, and 19 (1:25 dilution, 130-118−964, Miltenyi Biotec, Bergisch Gladbach, Germany), in T-buffer with 0.5% BSA. The mixture was then incubated at room temperature in the dark.

After incubation, the sample was washed twice. Immediately before the first sorting, the sample was filtered through a cell strainer (40 µL pores) (pluriSelect Life Science, Leipzig, Germany), and centrifuged. After centrifugation, the cell pellet was resuspended in 350 µL of T-buffer containing 0.5% BSA.

The cancer cells were enumerated and isolated using the On-chip Sort system according to the manufacturer’s instructions. Briefly, the flow path was prewashed with T-buffer containing 0.5% BSA. Maximum volume per sorting was 350 µL; therefore, if the volume of stained sample obtained from whole blood after red blood cell lysis was 1 ml, the sample was divided into three portions of approximately 350 µl each and sorted using the same disposable microfluidic chip, for the first sorting. The sorted cells gated into cytokeratin-positive channels were collected. After the first sorting, 100 µL of T-buffer containing 0.5% BSA was added to the used microfluidic chip and targeted cells were collected. The collected sample was transferred to a new chip, and sample volume was increased to 350 µL followed by second sorting.

After the second sorting, the recovered samples were stained with Alexa Fluor 647-conjugated anti-human CD340 (erbB2/HER-2) antibody (1:25 dilution, ab225510, abcam, Cambridge, UK), phycoerythrin (PE)-conjugated Anti-Vimentin Mouse mAb (VI-RE/1) (1:25 dilution, ab49918, abcam), PE-Cy7-conjugated anti-CD45 Mouse mAb (HI30) (1:50 dilution, ab 155340, abcam), 0.05 µg/mL of Hoechst33342 (ab145597, abcam), and FITC- CK3-6H5 (1:25 dilution, 130-118−964, Miltenyi Biotec). After staining, the collected samples were transferred to a new chip, and the sample volume was increased to 350 µL followed by third sorting. Targeted cells were collected by gating them into nuclei- and cytokeratin-positive and CD45-negative channels. Then, 100 µL of T-buffer containing 0.5% BSA was added to the used chip and targeted cells were collected again. Sample volume was increased to 350 µL followed by fourth sorting. Targeted cells were collected again by gating them into nuclei- and cytokeratin-positive and CD45-negative channels. The fluorescence signal intensities of HER2, vimentin, and cytokeratin were evaluated at the fourth sorting stage. The CTC enrichment process is shown in Supplementary Figure S4**a**. CTCs were defined as nucleated cells that lacked CD45 and expressed cytokeratin (Supplementary Figure S4**b**).

Flow cytometry was performed using an On-chip Sort system (On-chip Biotechnologies). Details of this system have been described previously and on the manufacturer’s website^36,44^. The On-chip Sort system used in this study employed three excitation lasers as described previously^36^. Data analysis was performed using On-chip Sort software version 1.9.9 (On-chip Biotechnologies). For multi-color analysis, a fluorescence compensation matrix was generated using single-stained cell line samples and Easy Comp beads for each fluorophore. This matrix was then applied to correct fluorescence compensation in all clinical sample measurements.

### HER2 and EMT progression in CTCs

The HER2-positive gastric cell lines MKN7, GSU, and NUGC4 exhibited fluorescence signal intensities of 489, 200, and 76, respectively. In contrast, the HER2-negative gastric cell line KATOIII showed a signal intensity of 50. The intensity was measured using the On-chip Sort system (On-chip Biotechnologies). The details of this system have been described previously and on the manufacturer’s website^36,44^. The optimal threshold that minimizes misclassification was determined based on the intersection point of the two normal distributions between HER2-negative (KATOIII) and HER2-positive (NUGC4) cell lines as follows:

T = µ1σ2^2^ + µ2σ1^2^/σ1^2^ + σ2^2^.

HER2-negative (KATOIII): Mean µ1 = 50, Standard deviation σ1 = 23.7.

HER2-positive (NUGC4): Mean µ2 = 76, Standard deviation σ2 = 46.1.

HER2 overexpression in CTCs was defined as a signal intensity of HER2 fluorescence > 56 in the CTC membranes. The rate at which trastuzumab kills cancer cells is proportional to the logarithm of HER2 expression amounts in a cancer cell^55^.

In this study, EMT induction was assessed using the EMT-index, which quantitatively integrates the contrast between cytokeratin and vimentin expression. The EMT-index was calculated from the ratio of the fluorescence signal intensity of cytokeratin and vimentin: fluorescence signal intensity of vimentin/(fluorescence signal intensity of cytokeratin + fluorescence signal intensity of vimentin). The fluorescent label dye for cytokeratin was designated FITC, and the fluorescent label dye for vimentin was designated PE. As the ratio changed from 100% FITC to 100% PE with EMT progression in cells, the EMT-index was normalized and changed from zero to one.

### Whole-genome amplification

Sorted cells were transferred from the collection reservoir to 200 µL polymerase chain reaction tubes and centrifuged at 600 × g for 10 min at room temperature. After discarding the supernatant, less than 2 µL were left, which was the starting volume of the whole-genome amplification (WGA). WGA was performed using an Ampli1 WGA kit (Silicon Biosystems, Bologna, Italy) according to the manufacturer’s protocol. DNA concentrations were determined using a Qubit 2.0 Fluorometer (Thermo Fisher Scientific) and quality control checks of the products were performed using the Ampli1 QC Kit (Silicon Biosystems). Only 15 samples that tested positive for all four PCR products were deemed to contain successfully amplified genomic material suitable for mutation analysis.

### Oncomine tumor mutation load assay

Among the 27 patients examined, tumor tissue DNA was obtained from six patients, and CTC DNA was obtained from 15 patients to confirm the occurrence of somatic mutations. Some tissue samples were obtained via biopsy, resulting in insufficient DNA for genetic analysis. Other tissue samples collected from surgical specimens contained adequate DNA, but the DNA quality was poor and unsuitable for analysis. CTC DNA was obtained from 15 patients because only these samples tested positive for all four PCR products in QC assessments, ensuring that they contained successfully amplified genomic material suitable for mutation analysis. The samples were subjected to genomic analysis after WGA. Library preparation and sequencing were performed according to the manufacturer’s instructions (Thermo Fisher Scientific). In brief, WGA of DNA originating from CTC was used for target amplification by Oncomine™ Tumor Mutation Load Assay that covers 1.7 megabase (Mb) of 409 genes with known cancer associations (Thermo Fisher Scientific). After the target amplification, a different barcode adaptor was ligated to each library to sequence multiple libraries on a single chip. The adaptor-ligated libraries were purified using Agencourt™ AMPure™ XP Reagent (Beckman Coulter). The concentration of each library was determined by qPCR using an Ion Library TaqMan^®^ Quantitation Kit (Thermo Fisher Scientific), and the dilution factor was calculated for each library. Multiple barcoded libraries were combined and sequenced using the Ion 540^TM^ Chip, Ion 540^TM^ kit-Chef, and Ion GeneStudio^TM^ S5 XL System (Thermo Fisher Scientific). Quality control and mapping of the sequencing reads were performed using Torrent Suite Software 5.12.2 (Thermo Fisher Scientific). Variant calls were performed using the Oncomine Tumor Mutation Load - w2.0 – DNA – Single Sample workflow in Ion Reporter Software 5.10 (Thermo Fisher Scientific). Annotations of the variants were based on dbSNP151, CCDS (NCBI, Release 15), RefSeq (UCSC Genoome Browser, Nov 2018), Gencode (UCSC Genome Browser, ver. 19), and 1000 Genomes (phase3 release v5). Genomic analysis was performed on selected 53 gene mutations, including GC driver mutations and EMT-related gene mutations, among 409 genes with known cancer associations^56,57^.

### Statistical analysis

All statistical analyses were performed using EZR version 1.31 (Saitama Medical Center, Jichi Medical University, Saitama, Japan) and JMP^®^11 (SAS Institute Inc., Cary, NC, USA)^58^. We also used R version 3.1.1 (The R Foundation for Statistical Computing, Vienna, Austria) as the graphical interface. The chi-square test was used to examine the associations between the three categorical variables. Continuous variables such as CTC number, EMT-index, and HER2 fluorescence signal intensity in CTCs, were evaluated using Shapiro-Wilk statistics, which showed that they were not normally distributed or homoscedastic. Thereafter, the Spearman’s correlation coefficient was used. EMT-index were compared using the Kruskal-Wallis test. PFS was measured to assess the prognosis. PFS was defined as the time from the start of chemotherapy to the confirmation of progression based on initial radiological findings. PFS curves were constructed using the Kaplan–Meier method.

## Electronic supplementary material

Below is the link to the electronic supplementary material.

Supplementary Material 1

## Data Availability

The datasets generated and/or analysed during the current study are available in the Sequence Read Archive (SRA) repository, (Accession code: PRJDB19600).
