# Supplementary material for: Real-time assessment of circulating tumor cells refines the indication for HER2-targeted therapy in metastatic gastric cancer
Source: Sci Rep. 2025 Jul 4;15:23906. doi: 10.1038/s41598-025-06913-x (PMC12227603; doi:10.1038/s41598-025-06913-x)
Supplement: Supplementary file 1 — Supplementary Material 1 [file 41598_2025_6913_MOESM1_ESM.pdf]

Supplementary Information for: Real-time assessment of circulating tumor cells refines the indication for HER2-targeted therapy in metastatic gastric cancer.

Yasuaki Kimura[1], Koichi Suzuki[1], Sawako Tamaki[1], Iku Abe[1], Yuhei Endo[1], Kosuke Ichida[1], Yuta Muto[1], Fumiaki Watanabe[1], Masaaki Saito[1], Kazuo Takeda[2] and Toshiki Rikiyama[1]

Supplementary Information:

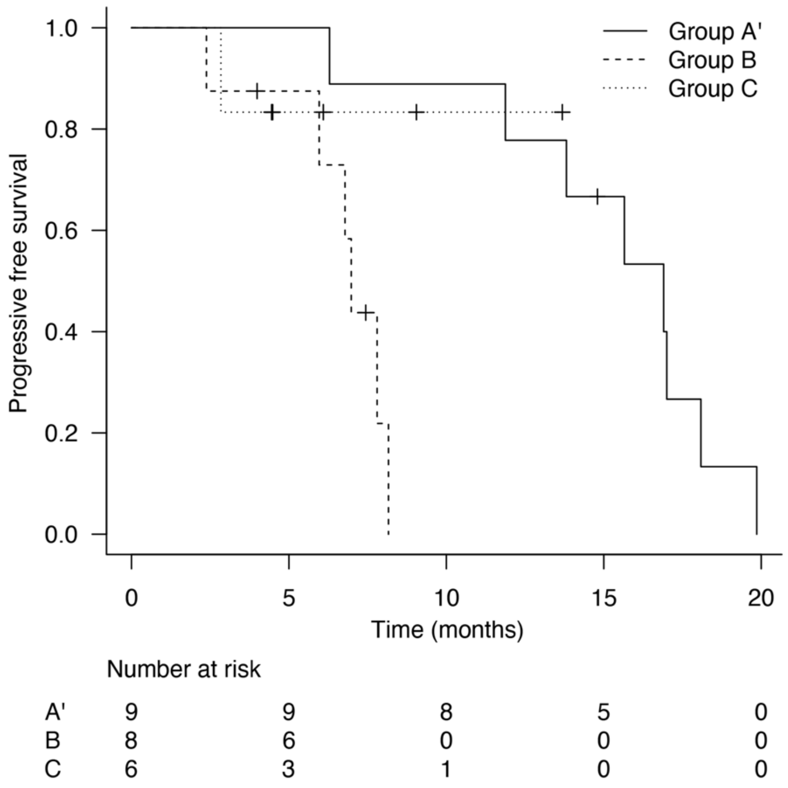

Supplementary Figure S1. Comparison of progression-free survival between the three groups in 23 patients.

The median PFS for groups A' (9 patients with CTC detection who were assigned to group A), B, and C was 16.9 months (95% CI, 6.29 to 18.1), 7.0 months (95% CI, 2.38 to NA), and

not reached (95% CI, 2.84 to NA), respectively (p=0.008). PFS, progression-free survival; CI, confidence interval; NA, not available

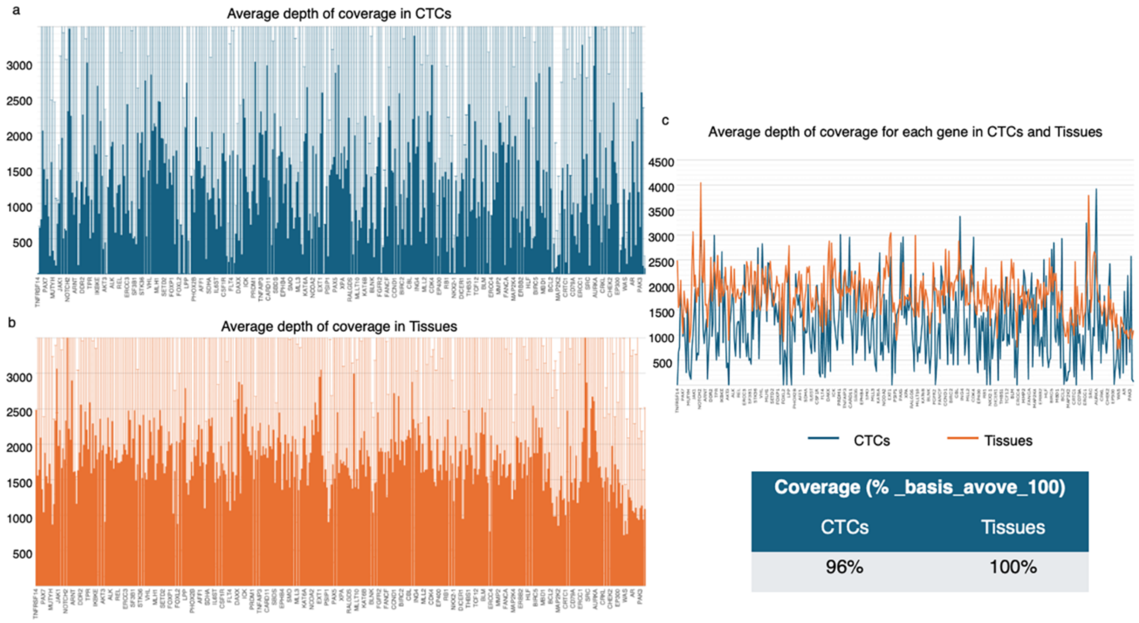

Supplementary Figure S2. Average depth of coverage for each gene in six CTCs and tumor tissue samples. The average depths of coverage in six CTC samples and six tumor tissue samples were compared. Figure S2a displays the average depth of coverage in CTC samples, Figure S2b shows the average depth of coverage in tumor tissue samples, and Figure S2c presents a comparison of the average depth of coverage between CTC and tumor tissue samples. In Figures S2a–S2c, gene names are listed on the horizontal axis, and the average depth of coverage is represented on the vertical axis. The figures illustrate the average depth of coverage for 409 genes; however, due to space limitations, only a subset of genes is shown. Additionally, in Figures S2a and S2b, the maximum displayed average depth of coverage was set to 3,500 to improve data visualization. The percentage of bases covered by at least 100 reads (%_bases_above_100) was calculated using data obtained from the average depth of coverage.

Landscape of genomic alterations in all 53 genes.

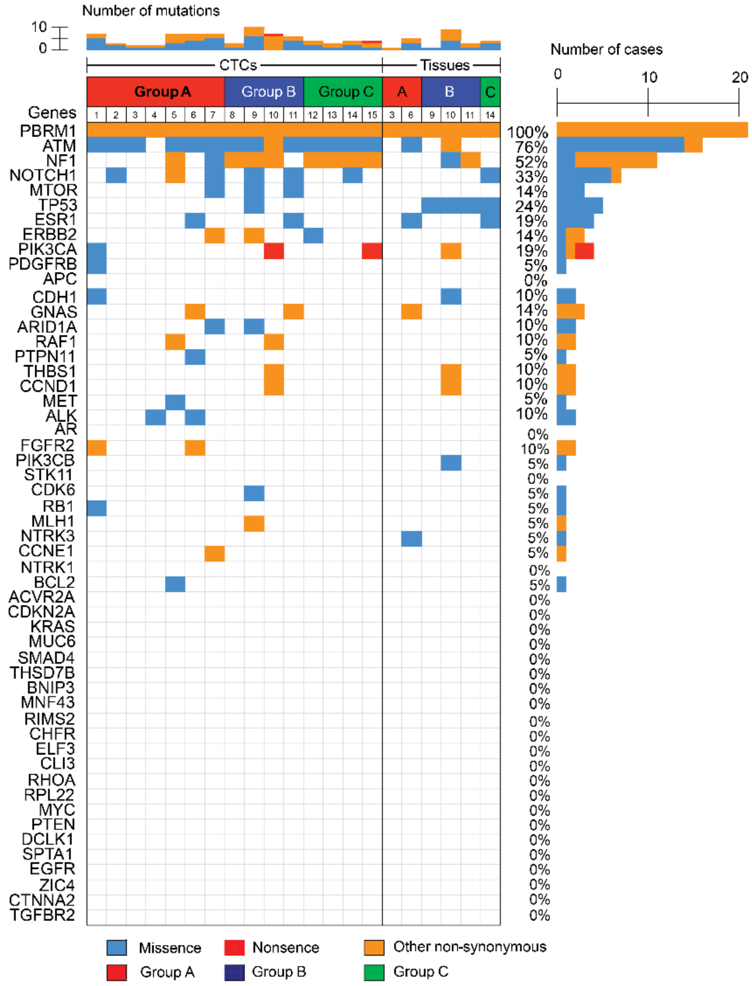

Supplementary Figure S3. Landscape of genomic alterations in all 53 genes.

The heat map on the left shows the genomic change landscape of the CTC samples, and that on the right shows the genomic change landscape of the tissue samples. The number of cases (%) is shown in the heatmap. Genomic alterations were compared among groups A, B, and C. Patients are ordered by case number, and identical numbers indicate samples from identical patients. The colors indicate specific types of genomic alterations, including missense mutations (blue), nonsense mutations (red), and other nonsynonymous mutations (orange). Each row indicates a gene, and each column represents a sample.

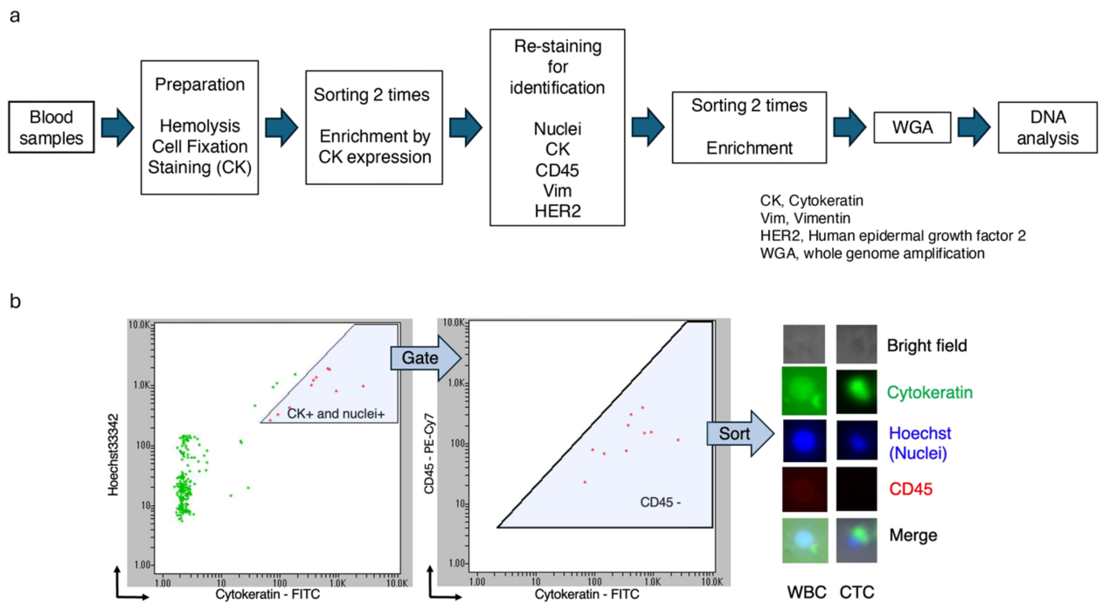

Supplementary Figure S4. Flowchart of CTC enrichment process, sorting gates for CTCs, and representative images of CTCs and WBC. Figure S4a shows CTC enrichment process for gastric cancer patients, including CTC counting, HER2 expression and EMT analysis. Additionally, purified CTCs were collected for each patient, followed by WGA processing for DNA sequencing analysis. Figure S4b shows sorting gates for CTCs and representative images of CTCs and WBC. CTCs gated as cytokeratin+/nuclei+/CD45- were sorted. After sorting, fluorescence compensation and protein expression analysis were performed. Nuclei, cytokeratin and CD45 staining was used to distinguish CTCs (CTC; nuclei+/CK+/CD45-) from the white blood cell population (White blood cell, WBC; nuclei+/CK+/CD45+). CTCs, circulating tumor cells; EMT, epithelial mesenchymal transition; WGA, whole genome amplification

Supplementary Table S1. Histological HER2 status determined by IHC and FISH in 27 patients with metastatic gastric cancer.

Case	Histological HER2 status	HER2 IHC score	FISH
1	Negative	0	ND
2	Negative	0	ND
3	Negative	0	ND
4	Negative	0	ND
5	Negative	0	ND
6	Negative	1+	ND
7	Negative	2+	Negative
8	Negative	2+	Negative
9	Negative	2+	Negative
10	Negative	2+	Negative
11	Negative	2+	Negative
12	Negative	2+	Negative
13	Negative	2+	Negative
14	Negative	2+	Negative
15	Positive	2+	Positive
16	Positive	2+	Positive
17	Positive	2+	Positive
18	Positive	3+	ND
19	Positive	3+	ND
20	Positive	3+	ND
21	Positive	3+	ND
22	Positive	3+	ND
23	Positive	3+	ND
24	Positive	3+	ND
25	Positive	3+	ND
26	Positive	3+	ND
27	Positive	3+	ND

HER2, human epidermal growth factor receptor 2; IHC, immunohistochemistry; FISH, fluorescence *in situ* hybridization; ND, not determined.

Supplementary Table S2. Comparison of clinicopathological features between the three groups in 23 patients with metastatic gastric cancer, based on HER2 status in tumors and CTCs

Total (n = 23)	Group A' n = 9 (%)	Group B n = 8 (%)	Group C n = 6 (%)	P value
Sex, male	8 (88.9)	8 (100)	3 (50)	0.041
Age, > 71 years	6 (66.7)	4 (50)	3 (50)	0.734
ECOG PS, 1	7 (77.8)	6 (75)	4 (66.7)	0.888
Disease status				
Initially metastatic	8 (88.9)	3 (37.5)	6 (100)	0.013
Recurrent	1 (11.1)	5 (62.5)	0 (0)	0.013
Metastatic or recurrent sites				
Anastomotic/Remnant	0 (0)	3 (37.5)	2 (33.3)	0.039
Peritoneum	6 (66.7)	3 (37.5)	2 (33.3)	0.041
Liver	4 (44.4)	2 (25)	2 (33.3)	0.171
Distant lymph nodes	4 (44.4)	3 (37.5)	5 (83.3)	0.198
Other sites	1 (11.1)	7 (87.5)	0 (0)	0.676
Bormann, type 4	0 (0)	1 (12.5)	2 (33.3)	0.171
Histology, undifferentiated	1 (11.1)	7 (87.5)	5 (83.3)	0.002
HER2 status, positive	9 (100)	0 (0)	0 (0)	< 0.001

Data are presented as n (%). ECOG PS, Eastern Cooperative Oncology Group performance status; HER2, human epidermal growth factor receptor 2; CTC, circulating tumor cell; group A', group A' included 9 patients with CTC detection who were assigned to group A
